# Asymmetric arms races between predators and prey: a tug of war between the life–dinner principle and the rare-enemy principle

**DOI:** 10.1098/rspb.2024.1052

**Published:** 2024-10-09

**Authors:** Donald James McLean, Marie E. Herberstein, Hanna Kokko

**Affiliations:** ^1^ School of Natural Sciences, Macquarie University, North Ryde, New South Wales, Australia; ^2^ Leibniz Institute of the Analysis of Biodiversity Change, Hamburg 20146, Germany; ^3^ Institute of Animal Cell and Systems Biology, University of Hamburg, Hamburg 20148, Germany; ^4^ Department of Evolutionary and Environmental Studies, University of Zurich, Zurich 8057, Switzerland; ^5^ Institute of Organismic and Molecular Evolution, Johannes Gutenberg University of Mainz, Mainz 55128, Germany; ^6^ Institute for Quantitative and Computational Biosciences, Mainz 55128, Germany

**Keywords:** predator–prey theory, anti-predatory behaviour, arms race, life–dinner principle, natural enemies

## Abstract

Antagonistic co-evolution can be asymmetric, where one species lags behind another. Asymmetry in a predator–prey context is expressed by the ‘life–dinner principle’, a classic informal model predicting that prey should be in some sense ahead in this arms race, since prey are running for their lives, while predators lag as they only run for their dinner. The model has undergone surprisingly little theoretical scrutiny. We derive analytical models that show coevolutionary outcomes do not always align with the life–dinner principle. Our results show that other important asymmetries can easily reverse the outcome, especially the rare-enemy principle: predators are usually outnumbered by their prey, sometimes substantially (trophic asymmetry), which can make selection on prey relatively weak. We additionally show that the antagonists typically exhibit different evolutionary responses to a situation where both predator and prey start out as equally fast runners. Although predators sometimes become so efficient that attacks always succeed, attack success often reaches a stable intermediate value. We conclude that the life–dinner principle has some validity as a metaphor, but its effect is of an ‘all else being equal’ type, which is surprisingly easily overridden by other features of the evolutionary dynamics.

## 1. Introduction

Evolutionary arms races refer to situations involving adaptations and counteradaptations of at least two parties (e.g. species or two sexes within the same species). Interspecific examples involve parasites and hosts (with consequences for infection dynamics [1,2]), interactions between brood parasitic birds and their hosts [3–5], and predator–prey interactions [6–8]. Since arms races predict, for individuals of each species, that gains could be made at the expense of individuals of the other species (escalatory Red Queen dynamics *sensu* Brockhurst *et al*. [9]), a key unresolved question is what factors might favour one party gaining an advantage over the other.

A classic prediction highlights the possibility of a ‘built-in imbalance between predator and prey with respect to the penalty of failure’, to use the phrase of Dawkins & Krebs [10]. This is their ‘life–dinner principle’, where the relevant mental image is that one individual is running for its life, another only for its dinner, and the outcome matters less for the latter. All else being equal, the asymmetric cost of being the loser (death versus missing one meal) is assumed to translate into stronger selection on prey than predators, yielding the prediction that—all else being equal—defensive adaptations will be more effective than offensive adaptations [10]. The principle continues to be referenced as a mechanism to explain low success rates of predators (e.g. [11,12]). In a related field of mutualistic or antagonistic symbioses, however, there is increasing recognition that all else is not necessarily equal: in addition to what they call ‘differences in the importance of the interaction’, Bergstrom & Lachmann [13] list differences in generation time, population size, or amount of segregating genetic variation as factors impacting the final outcome (and in this context slower evolutionary rates may prove beneficial to a participant in the game; see also [14]).

The life–dinner principle itself has been criticized on a number of grounds (briefly reviewed in [15]). Factors thought to work against the life–dinner principle include the possibility that predator–prey co-evolution is unlikely to occur at all unless prey are dangerous or lethal [16]; some anti-predator adaptations, such as aggregations, can increase predator success rates, precluding an arms race [17]; feedback between predator and prey adaptations may take more diverse forms than positive feedback [18]; stronger selection on prey may lead to reduced heritable variation, hence reducing the rate of prey trait evolution [16]; and improved anti-predator defences may lead to increased prey densities, so increased prey encounter rates may compensate for reduced attack success rates [18]. Furthermore, Humphreys & Ruxton [15] demonstrated that low predator success rates can be explained by mechanisms other than the life–dinner principle. They modelled an alternative principle — the ‘dicey dinner principle’—whereby predators may call off an attack to reduce the chance of their own injury or death, leading to low success rates under some circumstances.

Here, we highlight more fundamental reasons why the life–dinner principle does not straightforwardly predict arms race outcomes, than the behavioural details discussed above. The issue is the following. It is undoubtedly true that the fitness outcome of a single encounter has an asymmetry: a successful attack is worse for the prey than failing is for the predator. Whether it follows that the evolutionary response is asymmetric (stronger selection on the prey, leading to predators lagging behind in an arms race) is a question that has escaped formal scrutiny. Dawkins & Krebs [10] are not always clear about the need to differentiate between the single-encounter asymmetry and the evolutionary consequence: when they write, ‘a lineage under strong selection may out-evolve a weakly selected one (‘the life–dinner principle’)’, they inadvertently encourage an interpretation where the evolutionary prediction is thought to be equivalent to the single-encounter asymmetry. We ask whether the evolved asymmetry in the arms race follows the intuition offered by the single-encounter premise (life–dinner principle) and show this to be the case under certain ecological conditions only—and not necessarily the most likely ones in nature.

We show, using two different models, that even if the premise of the life–dinner principle holds—that is, one individual is ‘running for its life’ while the other is ‘running for its dinner’ (the single-encounter asymmetry)—this does not automatically imply that selection on prey is stronger than that on predators, or that the arms race reaches a state where prey are consistently ahead. In many cases, the outcome is better predicted by the rare-enemy principle: abundant prey are unlikely to evolve substantially in response to rare predators [19, p. 65]. It is interesting to note that one of the authors of the life–dinner principle also proposed the rare-enemy principle, but the contrasting predictions of the two principles do not appear to have been formally incorporated into an analysis of arms races between predators and their prey.

In our first model, we derive the strength of selection on the ability of prey or predators to modify the attack success probability, without assuming anything about the underlying trait that enables prey to escape attacks or predators to succeed in making a kill. In the second model, we define a trait that is expressed in both species (conceptualized as running speed) and investigate how running speeds of prey and predator coevolve. The coevolutionary outcome allows us to predict the magnitude of the lag, which is easiest to conceptualize as an empirically measurable property: the attack success probability, which is 0.5 if neither species is ahead in the arms race, <0.5 if the prey are ahead and >0.5 if the predators are ahead.

## 2. Model 1: the importance of trophic asymmetries and deaths from other causes

Our first model focuses on the first reason why the life–dinner principle is not the sole determinant of arms race outcomes: the unequal population sizes of prey and their predators. Population densities decline with increasing trophic levels, and prey populations are expected to outnumber their predators, often substantially [20,21]. This trophic asymmetry creates the ‘rare-enemy principle’ (i.e. an asymmetry in the frequencies with which a specific individual takes part in an encounter between predators and prey).

The importance of predation avoidance for the prey depends on how often predation, as opposed to other causes, could end an individual’s life. The other causes may relate to disease, injury, but also other types of predators, if their techniques of capturing prey are different and thus do not select for the same trait as we assume to be important for avoiding attack by a focal predator species. For simplicity, we therefore use the term ‘selective deaths’ for deaths causally related to the predator-prey interaction and non-selective deaths for all other causes. To make the argument complete, we also derive the number of selective and non-selective deaths of predators.

Our first model assumes, for simplicity, that fitness is proportional to lifespan (we thus do not explicitly model e.g. juvenile life stages during which no reproduction occurs yet). We use subscripts x and y for prey and their predators, respectively. Both species experience species-specific baseline mortality (causing non-selective deaths) and we denote the baseline mortality rate by μ_x_ for prey and μ_y_ for predators. For prey, the baseline refers to mortality that cannot be avoided even if predation attempts are always successfully avoided. For predators, the baseline refers to mortality that cannot be avoided even if prey encounters are always successful. The mortality terms μ_x_ and μ_y_ thus keep lifespans intrinsically finite for both species.

We derive fitness in a continuous-time setting [22], which means that mortalities can take any positive value (including values >1). When mortality occurs as a constant age-independent hazard, lifespans are exponentially distributed and mean lifespan is the reciprocal of mortality rate. The mortality rate can exceed 1 because this simply means an average lifespan of less than 1 unit of time [22]. To include the trophic asymmetry in the model, we consider that there are *N*_x_ prey and *N*_y_ predators and assume that typically *N*_x_ > *N*_y_ (but model predictions can also be derived for *N*_x_ ≤ *N*_y_). Populations are kept at constant size, and each of the *N*_y_ predators attacks prey at a rate of *a* per unit time (note that time units are the same for mortality and for attack rates). Each predator focuses on one randomly chosen prey at a time.

We assume populations do not diminish in size when finite lifespans come to an end. Although at first sight counterintuitive, this does not represent a logical flaw [14]. Population regulation can account for constant population size, by assuming that deaths create vacancies that are immediately replaced by offspring of those who are currently alive (a so-called Moran process). Offspring produced at times when no vacancies are available will fail to recruit and do not contribute to evolution. This simultaneously justifies the assumption that fitness is proportional to lifespan: only those who are alive can participate in producing recruits that fill vacancies caused by deaths.

Realized lifespans fall short of 1/μ_x_ and of 1/μ_y_ for prey and predators, respectively, because we assume mortalities increase for either party in case of non-ideal performance in an arms race setting. The goal of the analysis is to derive the proportional increase in lifespan (compared with conspecifics) that is achieved if an individual has a beneficial trait value that enables it to enjoy a better-than-average outcome in predator-prey encounters, where ‘average’ refers to its conspecifics. We denote the current average outcome as *p*, defined as the proportion of encounters that end up in prey capture. Here, we need to remember an asymmetry: *p* should change in a favourable direction for lifespan to increase, thus selection favours prey whose traits reduce *p*, while the opposite applies to the predator. Therefore, to determine whether selection is stronger on predators or prey, we compare –(d*W*_x_/d*p*)/*W*_x_ to (d*W*_y_/d*p*)/*W*_y_, where *W* denotes fitness. Fitness in model 1 refers to the expected lifespan, with equations that will be derived below.

From here on, the perspectives for prey and predators begin to differ, and we will first describe selection on the prey, before switching to the predator perspective.

### (a) Prey perspective

The mortality rate of prey due to predation depends on the rate at which individual prey are attacked and the probability that an attack leads to a kill. We have chosen a notation where *a* is the attack rate from the predator perspective, which necessitates deriving the prey perspective separately. If each of the *N*_y_ predators attack, at a rate *a*, one prey individual out of *N*_x_ possible choices, then each individual prey is attacked at rate $a\;N_{y}/N_{x}$ . Given our assumption that a proportion *p* of attacks leads to death (of prey), the all-causes death rate for prey is $\mu_{x}+ap\;N_{y}/N_{x}$ . The expected lifespan for prey is the reciprocal of that rate:


(2.1) $$W_{x}=\;\frac{1}{\mu_{x}+ap\frac{N_{y}}{N_{x}}}.$$

Since we assume a perfect correlation between longevity and fitness, selection can be quantified as the increase in lifespan (relative to the current mean ${\bar{W}}_{x}$ , based on the current value of *p*) when *p* decreases,


(2.2) $$S_{x}=-\frac{\frac{\partial W_{x}}{\partial p}}{{\bar{W}}_{x}}=\frac{aN_{y}}{\mu_{x}N_{x}+apN_{y}}.$$

### (b) Predator perspective

Selection on predators follows a similar logic to the above, but with the key aspect of the ‘life–dinner principle’ incorporated: one failure in one predator–prey encounter does not necessarily mean that the predator dies. Predators, like prey, have two mortality components, but the structure of the second component is different. Predators have baseline mortality (non-selected deaths), denoted μ_y_, and they also may die due to starvation resulting from persistent failures in hunting success. ‘Falling behind’ in the arms race implies low foraging success (low value of *ap*), which increases mortality in a gradual fashion, with its steepness controlled by a parameter *b*. For simplicity, we call *b* sensitivity to starvation, but note that the proximate cause of death may relate to other problems that are created by low conditions (e.g. more risk taking during foraging [23]), not necessarily acute starvation.

When they formulated the life–dinner principle, Dawkins & Krebs [10] accepted that multiple predator foraging failures would eventually lead to starvation, but reasoned that it would not affect the outcome of the principle, since ‘foxes who often fail to catch prey eventually starve to death, but they may get some reproduction in first’ [10].

How mortality responds to low foraging success could, in principle, take many functional forms. Here we will assume that starvation mortality increases in proportion to the reciprocal of foraging success, such that all-cause mortality for the predator is $\mu_{y}+b/ap$ . This form has the desirable property that the mortality cost of failure increases nonlinearly as foraging success drops: predator mortality is relatively insensitive to the value of *p* when *p* is at the high end of its possible range (where most attacks succeed) and much more sensitive to *p* when *p* is low. Low values of *b* indicate that predators are not particularly sensitive to failures (i.e. can fail more often before starving to death). The expected lifespan of predators is now


(2.3) $$W_{y}=\;\frac{1}{\mu_{y}+\frac{b}{ap}},$$

and thus, the strength of selection on predators takes the form


(2.4) $$S_{y}=\frac{\frac{\partial W_{y}}{\partial p}}{{\bar{W}}_{y}}=\frac{b}{bp+\mu_{y}ap^{2}}.$$

Assuming that background mortality is positive for both predators and prey (i.e. non-selected deaths are possible), we can use equations (2.2) and (2.4) to determine when selection is stronger on prey than predators. This is the essence of the life–dinner principle, and it is fulfilled when


(2.5) $$b<{\left(ap\right)}^{2}\frac{N_{y}}{N_{x}}\frac{\mu_{y}}{\mu_{x}}.$$

Whenever inequality (2.5) is satisfied, the prediction made by the life–dinner principle is supported by formal modelling: selection is stronger on prey than on predators.

A low value of *b* implies that lives of predators are not easily ended through starvation, while higher values imply that predators are very sensitive to foraging failures. Since inequality equation (2.5) states that the former condition (low *b*) must be true for selection to be stronger on prey than predators, there is a sense in which equation (2.5) simply recovers the key idea behind the life–dinner principle. However, this does not extend to equation (2.5) also proving the principle’s validity as a predictor of asymmetries in selection strength. Since both sides of the inequality are positive in equation (2.5), it is not *a priori* clear which side takes the greater value and thus, how easy it is for equation (2.5) to be satisfied. It is, therefore, of interest to examine the right-hand side of equation (2.5) in more detail.

A high number of attacks (attempts) per time unit per predator, *a*, that combines with a high *a priori* probability that an attack leads to capture, *p*, makes it easier for the outcome to align with the life–dinner principle (the range of *b* values where this is true becomes larger). A high number of predators *N*_y_ and high predator baseline mortality μ_y_ have a similar effect. These should combine with a low number of prey individuals, *N*_x_, and a low baseline mortality of prey, μ_x_, to find the most favourable conditions for selection to be stronger on prey than predators. The fact that the threshold value for *b* for equation (2.5) to be satisfied depends on no fewer than six other parameters, shows there to be potential for much variation in how predator-prey arms races play out.

**Figure 1.**
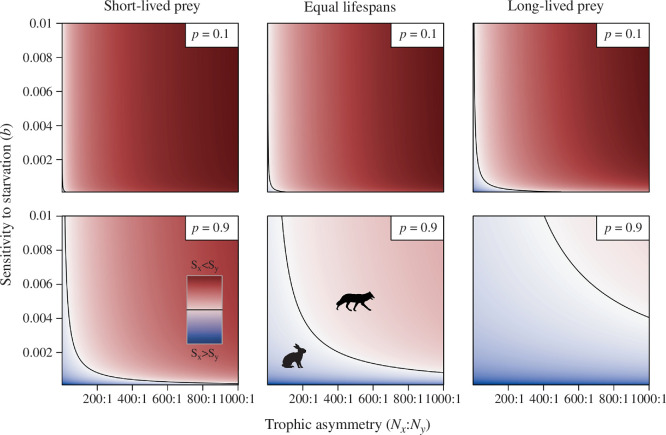
Heatmap showing the ratio of strength of selection on prey (*S*_x_, equation (2.2)) to predators (*S*_y_, equation (2.4)) for a range of parameter values. The life–dinner principle drives the outcome in lower-left regions of each panel (*S*_x_ > *S*_y_; blue), while the rare-enemy principle overrides the life–dinner in the upper-right regions (*S*_x_< *S*_y_; red). The black contour line indicates no difference in selection strength. Population ratio, *N*_x_:*N*_y_, varies along the *x*-axis; predator sensitivity to starvation, *b*, varies along the *y*-axis; mortality ratio µ_x_/µ_y_ = 5, 1 and 1/5 in the left, central and right columns, respectively; *a* = 1; predator success rate, *p*, as indicated.

It is particularly interesting to note that equation (2.5) predicts the life–dinner principle would be most easily applicable (i.e. prey experience stronger selection than predators across a large range of values for *b*) when there are few prey and many predators. However, ecological principles predict prey to be more abundant than their predators [20]. This implies that each combination of parameters has a prey availability beyond which selection becomes stronger on the predators than on the prey (in figure 1, moving towards increasing values of the trophic asymmetry, *N*_x_:*N*_y_, shifts the solution from blue, where the outcome aligns with the life–dinner principle, to red, where it does not). Another perspective on the same problem is the statement that for each value of the trophic asymmetry, there will be a threshold value of starvation sensitivity, *b*, above which the outcome no longer aligns with the life–dinner principle because inefficient predators have much-reduced lifespans compared to conspecifics with better hunting success. Also, other factors override the life–dinner principle more easily if the current success of predators is meagre (low *p*, figure 1).

A special case occurs when there are no non-selected deaths (μ_x_ = μ_y_ = 0) so that we assume that the only reason for prey to die is via encounters with the predator, and likewise the only reason for a predator to die is starvation. Instead of trusting equation (2.5), which in this setting divides by zero, the analysis must be repeated by computing the strengths of selection (equations (2.2) and (2.5)) with μ_x_ and μ_y_ set to 0, which yields *S*_x_ = *S*_y_ = 1 */p*. In other words, if nothing else kills them, selection will not differ between predators and prey—regardless of the modelled sensitivity of predators to starvation (value of *b*). While at first sight counterintuitive, this result gives an interesting insight into the symmetries in the situation. If *b* is low, predators are relatively long-lived to begin with, and a substantial increase in lifespan must be achieved by an individual for it to have substantially improved fitness. If *b* is high, predator lifespans are shorter, but so is the requirement for a lifespan improvement for this to represent a certain magnitude of improved fitness. Both can be achieved by the same improvement to a baseline hunting success, and selection is stronger if the current success rate (*p*) is low.

## 3. Model 2: Explicit traits and arms race dynamics

Above, we derived the strength of selection acting in either the prey or a predator population to change attack success *p*, but without commenting on how this could be achieved with actual traits. We also did not quantify the time scale over which an evolutionary response should be measured. Given that predator and prey lifespans, and thus generation times, are likely different, it is important to consider trait changes over an equivalent (absolute) time scale. In a related field of mutualistic interactions, it has been pointed out that evolutionary rates can have counterintuitive effects: the more slowly evolving species may gain a disproportionate share of the benefits that are at stake (Red King effect *sensu* Bergstrom & Lachmann [13]).

Later work has shown that switching the angle from a mutualistic to an antagonistic symbiosis predicts that having a fast evolutionary rate is always advantageous [14]. Predator–prey interactions are clearly antagonistic, and intuition suggests that the one with shorter generation times might evolve to be ‘ahead’ in an arms race, as there needs to be a shift from the parental generation to that of offspring for the response to selection to be fully visible. However, how this plays out in the case of overlapping generations is not clear based on intuition alone [13,14]. In this section, we derive numerical examples that show that either species can be ahead, and this does not necessarily align with being the one with the shorter generation time.

We use running speed as the conceptual example of a trait that determines capture probability. We will distinguish between *speed*, which is a trait of individuals, and *pace*, by which we mean a rate of evolutionary change over a certain amount of absolute time. We assume that the populations consist of individuals whose speeds are normally distributed with means $\overset{-}{x}$ and $\overset{-}{y}$ and variance 1:


(3.1a) $$x∼N\left(\overset{-}{x},1\right)$$

(3.1b) $$y∼N\left(\overset{-}{y},1\right)$$

We assume that in an encounter between a prey with speed *x* and a predator with speed *y*, the faster participant wins. The population-wide probability of a kill is therefore $p\left(\overset{-}{x},\overset{-}{y}\right)=P\left[y>x|\left\{x∼N\left(\overset{-}{x},1\right),y∼N\left(\overset{-}{y},1\right)\right\}\right]$ , and it equals the proportion of the bivariate normal distribution with means $\overset{-}{x}$ and $\overset{-}{y}$ that lies above the diagonal $\bar{y}\;=\;\bar{x}$ .


(3.2) $$p\left(\overset{-}{x},\overset{-}{y}\right)=\int_{-\infty }^{\infty }\int_{\overset{-}{x}}^{\infty }\frac{1}{2\pi }e^{-\;\frac{{\left(x-\overset{-}{x}\right)}^{2}+{\left(y-\overset{-}{y}\right)}^{2}}{2}}\;dy\;dx$$

Equation (3.2) has the shape of the cumulative normal distribution, where identical mean speeds $\bar{y}\;=\;\bar{x}$ predict *p* = 0.5. Equation (3.2) can be used to answer a population-wide question: how much does *p* change when the prey (or the predator) population changes its speed somewhat (i.e. what are the values of $\partial p/\partial \overset{-}{x}$ and $\partial p/\partial \overset{-}{y}$ ). Using the Leibniz integration rule, we find the solutions


(3.3a) $$\frac{\partial p}{\partial \overset{-}{x}}=-\frac{1}{2\sqrt{\pi }}e^{-\;\frac{{\left(\overset{-}{x}-\overset{-}{y}\right)}^{2}}{4}}$$

(3.3b) $$\frac{\partial p}{\partial \overset{-}{y}}=\frac{1}{2\sqrt{\pi }}e^{-\;\frac{{\left(\overset{-}{x}-\overset{-}{y}\right)}^{2}}{4}}$$

The identical form (but opposite sign) of (equation 3.3a,b) has clear implications for the dynamics: all else being equal, selection is strongest on both parties when predator and prey speed distributions overlap maximally (i.e. when $\overset{-}{x}=\overset{-}{y}$ ) and weaken when the speeds begin to differ. In other words, when one species is clearly ‘ahead’ with a superior trait value, selection becomes weaker not only for the faster species, but, perhaps counterintuitively, also for the slower one. This is easiest to understand by considering the case where speeds are almost completely non-overlapping: if, for example, predators are much faster than prey so that kills almost invariably succeed, then small improvements in prey speed are hardly rewarded. Under such a situation, prey simply reproduce until a predator finds them, and luck (of not being found for a while) determines the lifespan of prey.

Although there is a symmetry of the form $\partial p/\partial \overset{-}{x}=-\;\partial p/\partial \overset{-}{y}$ (which is true when the populations do not differ in their variances), we have already learned (model 1) that the importance of *p* itself for individual fitness, and hence selection, may differ between predators and their prey. This raises the next question: if one species at present has a faster-paced evolutionary response than its antagonist, will this pace difference continue into the future, or does the pace slow down as the speed difference between the two parties increases? If the former is true, i.e. an initial asymmetry in responses to selection extrapolates far into the future too, then one of the two antagonists evolves to win the arms race completely such that *p* = 0 or *p* = 1 are the only possible outcomes. If the latter is true, then an arms race may reach a phase where the speed difference between both species has stabilized to some intermediate value, and even if the speeds themselves change over time, they do so at the same pace: the speed difference, and hence the value of *p*, remains constant thereafter.

**Figure 2.**
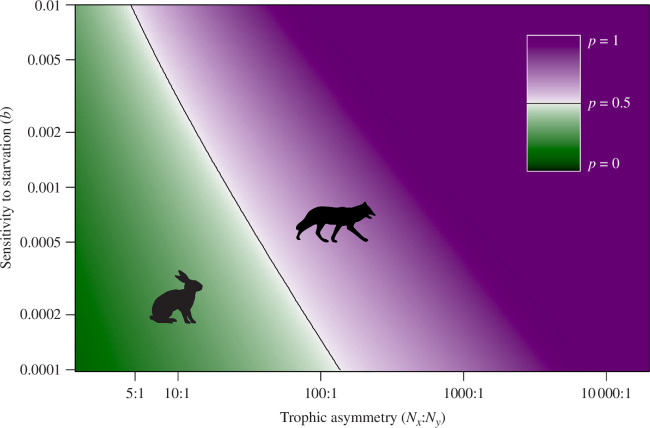
Arms race outcome across a range of parameters, predicted using model 2. Colours indicate the lag, measured as the predator’s success rate (p) when prey and predators are both evolving at the same pace. When *p* < 0.5, prey are ahead; the life–dinner principle is the dominant determinant of the outcome (green region with rabbit symbol). When *p* > 0.5, predators are ahead; the rare-enemy principle dominates the outcome (purple region with fox symbol). Black contour line indicates the boundary between the two regions (*p* = 0.5). Trophic asymmetry varies along the *x*-axis, *b* along the *y*-axis, µ_x_ = 1, µ_y_ = 1.

A numerical analysis of the model (see electronic supplementary material for details) shows that the latter is often true. When the trophic asymmetry is sufficiently strong (right end of figure 2), the arms race yields a constant value of *p* > 0.5, implying that predators are ahead in the arms race as they are, on average, the faster species when the trait is conceptualized as running speed ( $\overset{-}{y}>\overset{-}{x}$ , which implies *p* > 0.5 in equation (3.2)). Low trophic asymmetry (left end of figure 2) predicts, on the other hand, that prey are ahead, and attacks are rarely successful (*p* < 0.5). The boundary between these two situations (black line, figure 2) reflects the point where the life–dinner principle and the rare-enemy principle exactly cancel out. For sensitive predators (high *b*), a lower trophic asymmetry is sufficient for the rare-enemy effect to cancel out the effects of the life–dinner principle (figure 2: the black line ranges from high *b* and a low value for the trophic asymmetry to low *b* and a higher value of the trophic asymmetry).

There is an additional asymmetry in the model outcome. With a sufficiently strong trophic asymmetry, it is easy to find cases where predator–prey encounters are nearly always fatal for the prey ( p≈1 in the dark purple region of figure 2), but low trophic asymmetry does not yield a mirror image where *p* would evolve to be close to 0 (the darkest colours of the green colour scale for *p* are never reached in figure 2). This contrast reflects the importance of the rare-enemy principle. If attack success probability *p* is very low, predators are clearly selected to do better, and evolution is then expected to elevate *p*. But in situations where *p* is very high, so that predators are very successful in each encounter, it is still possible for prey not to experience very intense selection, as long as predators are rare relative to their prey (high trophic asymmetry).

## 4. Discussion

Traits that affect the outcome of predation have a large effect on fitness, so they are often thought to be under strong selection [24]. Dawkins & Krebs [10] argued that the lower cost of failure for predators reduces the strength of selection for offensive adaptations compared to defensive adaptations in prey, calling it the life–dinner principle. Not long afterwards, Dawkins [19] introduced the rare-enemy principle, reasoning that if predators are rare in relation to their prey, they will have little effect on the prey, so selection will be stronger on predators than prey. The contradiction between the life–dinner and rare-enemy principles has been noted previously [17,18,25], but these remarks do not appear to have led to any synthesis examining the relative roles of these verbal arguments in formal coevolutionary settings. Our models aim to fill in this gap.

Both of our models (figures 1 and 2) show that as predators become more resilient to failed attacks, selection on them weakens, as predicted by the life–dinner principle. However, the models also show that increasing the abundance of prey relative to predators decreases the strength of selection on prey to the extent that selection easily becomes stronger on predators than prey, counteracting the life–dinner principle. Since predators by definition occupy a higher trophic level than their prey, and this usually means that prey outnumber their predators, often substantially, blanket statements about the universality of the life–dinner principle appear misguided. Indeed, empirical evidence from research into the structure of trophic webs finds that foraging/consumer traits typically evolve much faster than resource/vulnerability traits [26–28], which contradicts a simplistic (‘blanket’) interpretation of the life–dinner principle.

We have followed Dawkins & Krebs [10] in modelling a single continuous trait (e.g. speed) to illustrate our argument. However, our arguments apply equally to other offensive and defensive traits that can co-evolve, with prey evolving to reduce the probability of being killed (the proportion of prey killed in attacks, *p*, in our analytical model) and predators evolving to increase the same quantity. Other potentially co-evolving traits could include chemical deterrents countered by an evolved tolerance in predators [29], or increasing size in bivalve shells countered by more accurately targeted attacks by predators [30].

There are many factors potentially affecting predator-prey interactions that were ignored by Dawkins & Krebs [10] and consequently by us. Our models produce open-ended arms races, so we did not model any kind of selective costs or trade-offs on the evolution of the trait (see [18]). In their informal model, Dawkins & Krebs [10] discussed a constraint derived from the cost of adaptation, but assumed it would not affect the *relative* speeds or strengths of selection on predators and prey. In nature, costs associated with success in an arms-race context may manifest as trade-offs that reduce selection for ever-increasing trait values, and asymmetries in such costs may impact arms-race outcomes. Further, our models avoid changes in population dynamics by assuming deaths are compensated for by reproduction, so population sizes remain fixed. We also omit details such as causal links between parameters *a* and *p* at the individual level: it is conceivable that a predator that fails may increase its attack rate. If such feedback were included in the model, the intuitive outcome is that the predators falling behind in the arms race (low *p*) would effectively appear more abundant to the prey as predators increase their *a*; this would mean that a value ‘more to the right’ in figures 1 and 2 becomes the correct prediction for a given trophic asymmetry, if the situation involves a low *p*. However, formal modelling is required to confirm this intuition, and this represents an interesting avenue for further work.

Our work also opens up other intriguing modelling possibilities. Compensatory predation (when predation replaces existing sources of mortality) may reduce selection on prey since prey killed by predators would die anyway before reproducing [31,32]. In addition, we assumed predators were unable to perceive the prey traits before attacking them. If predators preferentially target prey that are injured or sick, are young and inexperienced, or are old to the extent of being senescent—in all cases, easier to catch and overcome than normal prey—then the relevant values of attack success *p* change, and interact with prey life history traits (and selection on prey may diversify from a trait like ‘speed’ to underlying condition, e.g. via investment in immunocompetence). Spatial and density effects could also alter predator–prey interactions. For example, if prey exist at low spatial densities, encounter rates would be low so predators would be close to starvation and under greater pressure to succeed at each attack [33]. While some of these factors could fit within our models (e.g. low encounter rates would correspond with high values of *b* in the analytical model), others could have effects that are not addressed by earlier life–dinner models or by ours. Finally, we did not consider communities with multiple prey or predator species; anti-predatory traits that work well against one predator may be efficient in deterring other species to various degrees or not at all, with potentially interesting consequences for the strength of selection.

Trophic asymmetry is likely to have effects beyond the life dinner/rare enemy principles, so it may be valuable to take it into consideration whenever investigating interactions between trophic levels. For example, the ‘dicey-dinner’ principle reasons that asymmetrical costs of risk-taking during a single predator–prey encounter may lead to predators calling off an attack [15]. If we instead consider all attacks over an interval of time, trophic asymmetry means that individual prey will experience attacks less frequently than predators; hence, cumulative risk is higher for predators and optimal predator behaviour will be to accept lower per-attack risk than prey. Therefore, trophic asymmetry strengthens the ‘dicey-dinner’ principle. Trophic asymmetry should also be considered in contexts beyond predator–prey interactions, such as parasitism and mimicry, whenever the effect of selection by one party on another is modulated by their relative population sizes. Furthermore, trophic asymmetry is a special case of species-specific population sizes within a community of co-evolving populations. Extrapolating from our modelling, relative population size may be an important contributor to the strengths of selection within any co-evolutionary context, including mutualistic symbiosis.

As a whole, our models presented here provide limited support for the verbal argument by Dawkins & Krebs [10] that unequal cost of failure leads to unequal strength of selection, leading to prey ‘winning’ the predator/prey evolutionary arms race—the life–dinner principle. Instead, we show how one can integrate over several verbal arguments and examine the tug-of-war between counteracting forces (especially the rare-enemy principle, caused by trophic asymmetries). The consequence is a much richer diversity in the ways that relative population sizes and life history traits of predators and their prey modulate selection on predators’ foraging traits and the anti-predatory traits of their prey. While the life–dinner principle may lack generality as a predictive heuristic, our analysis shows numerous avenues for distinguishing between causalities: did a prey animal ‘win’ the race because it runs for its life, or ‘lose’ because it greatly outnumbers its predators and, loosely speaking, was consequently not quite expecting to encounter one so soon? Estimating the frequencies of different cases and linking them to the numerical asymmetries between predators and prey, as well as the food requirements of the predator, would be a fruitful task for empirical assessment of the underlying principles.

## Data Availability

Code (MATLAB and R scripts) used to create the figures in the article is included in the electronic supplementary material [34].
